# Identification and functional analysis of two oligopeptide transporters supporting the growth of Lacticaseibacillus paracasei strain Shirota in milk

**DOI:** 10.1099/mic.0.001624

**Published:** 2025-11-20

**Authors:** Shiho Tanaka, Masaki Serata, Tomohiko Terai, Daichi Fujii, Takekazu Okumura

**Affiliations:** 1 Microbiological Research Department, Yakult Central Institute, Kunitachi, Tokyo, Japan; 2 Basic Research Department, Yakult Central Institute, Kunitachi, Tokyo, Japan

**Keywords:** lactic acid bacteria, *Lacticaseibacillus paracasei *strain Shirota, oligopeptide-binding proteins, oligopeptide transporters

## Abstract

Oligopeptide transporters are important proteins in several lactic acid bacteria (LAB) that facilitate the transport of oligopeptides, the primary nitrogen source for growth in milk. Although the proteolytic mechanisms are well understood in some LAB species, limited research has been conducted on the peptide transport systems of *Lacticaseibacillus paracasei* (formerly *Lactobacillus casei*) strain Shirota (LcS), particularly its oligopeptide transporters. This study investigated the nitrogen uptake mechanism of LcS, a probiotic lactic acid bacterium, by generating gene knockout (KO) strains of two oligopeptide transporters, Opp_Lc_ and Dpp_Lc_. Consequently, the disruption of these genes eliminated the ability of the bacterium to grow in milk, identifying Opp_Lc_ and Dpp_Lc_ as the primary oligopeptide transporters in LcS. Growth in a leucine-free chemically defined medium with a Leu-containing peptide as the sole nitrogen source indicated that Opp_Lc_ and Dpp_Lc_ transport peptides of 4–8 and 3–7 residues, respectively. To our knowledge, this study provides the first experimental evidence of oligopeptide transporters in *Lactobacilli* capable of transporting peptides up to eight residues long. Analysis of KO strains targeting OppA_1_ or DppA_1_ to identify other oligopeptide-binding proteins (OBPs) within each oligopeptide transporter operon that may influence substrate specificity revealed that OppA_1_ is the only OBPs in Opp_Lc_. However, DppA_2_ and DppA_3_, encoded at chromosomal locations distant from the Dpp_Lc_ operon, may function as subunits constituting Dpp_Lc_ and DppA_1_. These findings enhance our understanding of nitrogen source utilization in lactobacilli and might inform future strategies to optimize nitrogen sources for LcS and improve culture technology for LcS-based products.

## Graphical abstract

*Lacticaseibacillus paracasei* strain Shirota (LcS) employs two distinct oligopeptide transporters, Opp_Lc_ and Dpp_Lc_, to facilitate nitrogen acquisition during growth in milk. These transporters exhibit differential substrate specificity, enabling the uptake of oligopeptides of varying lengths derived from milk proteins.

**Impact Statement**

Understanding how probiotic lactic acid bacteria (LAB) utilize nitrogen during milk fermentation is vital for improving the consistency and efficiency of dairy product manufacturing. This study advances the field by being the first study to identify multiple oligopeptide transporters operating within a single *Lacticaseibacillus paracasei* strain Shirota. These oligopeptide transporters are essential for importing peptides – key nitrogen sources – into bacterial cells. A novel insight from this study is that different transport systems exhibit distinct peptide preferences based on their associated binding proteins, highlighting the molecular diversity of peptide uptake strategies in LAB. This study provides a scientific understanding of nutrient acquisition in probiotic bacteria. It may also help develop future strategies to optimize bacterial growth and performance in dairy fermentations by tailoring nitrogen sources to match transporter specificity. Overall, this study offers an incremental yet important contribution to food biotechnology, laying the groundwork for more targeted manipulation of microbial function in fermented dairy products.

## Data Summary

The authors confirm that all supporting data and protocols have been provided within the article or through supplementary data files. The series of genes comprising *opp_Lc_* and *dpp_Lc_* was registered as a gene cluster in the DDBJ under accession numbers LC855157 and LC855158, respectively. The nucleotide sequences of *dppA_2_* (accession no. LC855159), *dppA_3_* (accession no. LC855160), *dppA_4_* (accession no. LC855161), *dppA_5_* (accession no. LC855162) and *dppA_6_* (accession no. LC855163) were also registered.

## Introduction

Lactic acid bacteria (LAB) obtain various aa from their environment that cannot be biosynthesized [1,2]. In milk, proteins are the primary nitrogen source for LAB owing to their limited free aa. To meet their nutritional requirements, LAB have developed proteolytic systems that hydrolyse milk proteins and absorb the resulting aa and peptides [3]. These processes have been well studied in dairy-associated strains, such as *Lactococcus lactis* [4,5], *Lactobacillus helveticus* [6,7], and *Streptococcus thermophilus* [8]. Despite species and strain differences, LAB typically process milk proteins through three main steps: (1) degradation of milk proteins into peptides by extracellular proteases, (2) transport of peptides – primarily oligopeptides – into bacterial cells and (3) breakdown of peptides into aa by intracellular peptidases [3].

The uptake of oligopeptides by LAB depends on oligopeptide transporters. In *L. lactis*, the most studied ABC oligopeptide transporter is the oligopeptide transport system (Opp system) [9,10], which includes the components OppA, OppB, OppC, OppD and OppF, each with specific functions in peptide transport. OppA is a membrane-associated substrate-binding protein, OppB and OppC form transmembrane domains and OppD and OppF are intracellular ATP-binding proteins. Although *opp* genes are conserved in lactobacilli [11], peptide transport characteristics vary among species and strains [12]. OppA initiates peptide transport via the Opp transport system and is a key for determining substrate specificity [13]. Studies on Opp-transported peptides have shown that OppA binds to various peptides, with 4–35 residue peptides as the primary targets of OppA in *L. lactis* [14]. Di- and tripeptides that cannot be transported by the Opp system are transported by the di-tripeptide transport system (Dpp system) [15], which includes five DppABCDF subunits and belongs to the same ABC transporter superfamily as the Opp system. Additionally, the *L. lactis* di-tripeptide transport protein (DtpT) system [16], which belongs to the peptide transport family, contains a single subunit and uses a proton concentration gradient for transport.

Variations in the number of oligopeptide-binding proteins (OBPs) in Opp and Dpp systems differ among species and strains [12]. *L. lactis* possesses only one OppA; however, some LAB have multiple copies of OBPs in their Opp systems. For example, the Ami transport system, which is analogous to the Opp system in *S. thermophilus*, uses three peptide-binding proteins as a single oligopeptide transporter [17]. In the *L. lactis* Dpp system, a second OBP, DppP, functions alongside DppA [15,18]. Among lactobacilli, only *Lactobacillus bulgaricus* has been shown to express two OppAs as operons [19]. Hence, the presence of multiple OBP is considered a microbial survival strategy to enhance transport efficiency in the peptide transport system, as it increases both the number of OBP molecules and the types of peptides that can be transported.

The molecular mechanism of peptide transport in lactococci, especially *L. lactis*, which is widely used in dairy production, has been thoroughly studied since the early 1980s, aided by advancements in genetic engineering [20]. However, much less is known about these mechanisms in *L. bulgaricus* and *L. helveticus*, despite their importance in yoghurt and cheese production. Furthermore, few studies have clarified the characteristics of their oligopeptide transporters. This scarcity could be due to the underexploration of efficient genetic engineering tools, especially for *L. bulgaricus*, which have only recently been established [21]. Nevertheless, genetic engineering approaches, including knockout (KO) strain generation, have been established for the *Lacticaseibacillus paracasei* strain Shirota (LcS), the strain used in this study [22]. Although *L. paracasei* is often isolated from non-dairy environments, such as the intestinal tract and oral cavity [23,24], LcS has a long history of use in dairy products, suggesting it may be uniquely adapted for growth in milk.

Given the limited functional analyses of peptide transport systems in lactobacilli, in this study, we aimed to investigate the nitrogen utilization mechanisms in LcS. Specifically, we sought to identify the key transport systems that facilitate growth in milk. Understanding these mechanisms will not only provide insights into the metabolic adaptations of LcS for dairy environments but also contribute to the broader understanding of LAB peptide transport systems. The findings may provide useful insights for optimizing dairy fermentation processes.

## Methods

### Bacterial strains

The strains and plasmids used in this study are listed in Table 1. *Escherichia coli* and *L. paracasei* strains were cultivated in Luria-Bertani medium or Difco Lactobacilli de Man, Rogosa, Sharpe (MRS) broth (BD Biosciences), respectively, and subsequently preserved at −80 °C. Frozen stock was used as the inoculum for subsequent experiments.

**Table 1.** Bacterial strains and plasmids used in this study

Strain or plasmid	Characteristic or relevant genotype	Source or reference
*E. coli*		
JM109	recA1, endA1, gyrA96, thi-1, hsdR17(rK^−^ mK^+^), e14^−^ (mcrA^−^), supE44, relA1, Δ (lac-proAB)/F' (traD36, proAB^+^, lacIq, lacZΔM15)	TaKaRa Bio
*L. paracasei*		
YIT 9029 (strain Shirota: LcS)	WT	Our collection
Δ*opp*	Opp_Lc_^−^ derivative of LcS	This study
Δ*dpp*	Dpp_Lc_^−^ derivative of LcS	This study
Δ*opp*Δ*dpp*	Opp_Lc_^−^ Dpp_Lc_^−^ derivative of LcS	This study
Δ*oppA_1_*	OppA_1_^−^ derivative of LcS	This study
Δ*opp*Δ*dppA_1_*	Opp_Lc_^−^ DppA_1_^−^ derivative of LcS	This study
Δ*opp*Δ*dppA_1_*Δ*dppA_2_*	Opp_Lc_^−^ DppA_1_^−^ DppA_2_^−^ derivative of LcS	This study
Δ*opp*Δ*dppA_1_*Δ*dppA_3_*	Opp_Lc_^−^ DppA_1_^−^ DppA_3_^−^ derivative of LcS	This study
Δ*opp*Δ*dppA_1_*Δ*dppA_4_*	Opp_Lc_^−^ DppA_1_^−^ DppA_4_^−^ derivative of LcS	This study
Δ*opp*Δ*dppA_1_*Δ*dppA_5_*	Opp_Lc_^−^ DppA_1_^−^ DppA_5_^−^ derivative of LcS	This study
Δ*opp*Δ*dppA_1_*Δ*dppA_6_*	Opp_Lc_^−^ DppA_1_^−^ DppA_6_^−^ derivative of LcS	This study
Δ*opp* Δ*dppA_1_*Δ*dppA_2_*Δ*dppA_3_*	Opp_Lc_^−^ DppA_1_^−^ DppA_2_^−^ DppA_3_^−^ derivative of LcS	This study
Δ*opp^C^*	Δ*opp* harbouring plasmid pYAP300-opp	This study
Plasmids		
pYSSE3	*E. coli* cloning vector carrying pUC19 *ori* region, pAM*β*1 erythromycin resistance gene and multi-cloning site	[22]
pYAP300	*E. coli* cloning vector carrying p15A *ori* region, pAM*β*1 erythromycin resistance gene, phiFSW *attP* site and *int* and multi-cloning site	[22]
pYSSE3-Δopp	pYSSE3 carrying upstream region with the N-terminus of *opp_Lc_* and the downstream region with the C-terminus of *opp*_Lc_	This study
pYSSE3-Δdpp	pYSSE3 carrying upstream region with N-terminus of *dpp_Lc_* and downstream region with C-terminus of *dpp*_Lc_	This study
pYAP300-opp	pYAP300 carrying *opp*	This study
pYSSE3-ΔoppA_1_	pYSSE3 carrying upstream region with N-terminus of *oppA_1_* and downstream region with C-terminus of *oppA_1_*	This study
pYSSE3-ΔdppA_1_	pYSSE3 carrying upstream region with N-terminus of *dppA_1_* and downstream region with C-terminus of *dppA_1_*	This study
pYSSE3-ΔdppA_2_	pYSSE3 carrying upstream region with N-terminus of *dppA_2_* and downstream region with C-terminus of *dppA_2_*	This study
pYSSE3-ΔdppA_3_	pYSSE3 carrying upstream region with N-terminus of *dppA_3_* and downstream region with C-terminus of *dppA_3_*	This study
pYSSE3-ΔdppA_4_	pYSSE3 carrying upstream region with N-terminus of *dppA_4_* and downstream region with C-terminus of *dppA_4_*	This study
pYSSE3-ΔdppA_5_	pYSSE3 carrying upstream region with N-terminus of *dppA_5_* and downstream region with C-terminus of *dppA_5_*	This study
pYSSE3-ΔdppA_6_	pYSSE3 carrying upstream region with N-terminus of *dppA_6_* and downstream region with C-terminus of *dppA_6_*	This study

### Sequence analysis and construction of gene engineering strains

Gene and aa sequence analyses, as well as promoter identification, were conducted using Genetyx version 14.1.0 (NIHON SERVER Co., Ltd.). Gene KO and genetic complementation strains were generated using a method described previously with slight modifications [25,26]. Briefly, KO strains were generated using PCR by synthesizing two gene fragments, each containing the upstream and downstream regions of the target gene. These fragments were subsequently ligated into the pYSSE3 vector using restriction enzymes or an In-Fusion HD Cloning Kit (TaKaRa Bio Inc.). Plasmids carrying the gene fragments were first introduced into the *E. coli* strain JM109 via electroporation. After amplification within the bacterial cells, the plasmids were extracted and subsequently introduced into the LcS WT or KO strains via electroporation. Single-crossover strains harbouring the gene of interest were then selected. Erythromycin-sensitive strains that emerged by homologous recombination in the process of repeated passaging of the selected single cross-over strains on medium without erythromycin were selected as KO strains (Fig. S1, available in the online Supplementary Material). To generate the *opp_Lc_* complemented strain (Δ*opp^c^*), the gene region containing the predicted Shine–Dalgarno and CDSs of *opp_Lc_* was cloned into the pYAP300 vector [22], designated as pYAP300-opp. This plasmid (pYAP300-opp) was then introduced into the *opp_Lc_* KO strain (Δ*opp*) via transformation. Erythromycin-resistant strains were selected using PCR for the presence of the *opp_Lc_* insert. The resulting strain was designated as Δ*opp^c^*. All primers used to generate the transgenic strains are listed in Table S1.

### Growth assays

To prepare the milk medium, skimmed milk powder (Megmilk Snow Brand) was reconstituted in water at a final concentration of 10% (w/v) and sterilized by autoclaving at 121 °C for 15 min. To evaluate the growth of each strain in the MRS medium, each frozen stock was inoculated into the MRS medium at 0.1% (v/v) and incubated for 24 h at 37 °C. The bacterial culture was transferred to fresh MRS medium under the same conditions and incubated for 24 h. For growth evaluation in milk, the inoculum was added at 0.1% (v/v) to a milk medium and incubated for 48 h. Each solution was diluted with 0.85% (w/v) saline to the appropriate concentration and spread on MRS agar plates using a spiral plate (EDDY JET2; IUL Instruments). The plates were incubated at 37 °C for 72 h, and the colony counts as c.f.u. were quantified using ProtoCOL3 (Synbiosis). Cumulative organic acid production by LAB during growth, an alternative indicator of bacterial proliferation, was assessed by measuring titration acidity. Each sample was diluted to the appropriate concentration in Milli-Q water, and the volume of 0.1 N NaOH needed to reach pH 8.5 was calculated per 9 g sample using an automatic acidity titrator (HIRANUMA Co., Ltd., Mito City, Japan). The percentage of lactic acid in a 9 g sample was calculated using the following formula:


Lactic acid (%)=(Titratable acidity×0.009)/9×100

### Transport test of leucine-containing peptides

Chemically defined medium lacking leucine [CDM (Leu−)] was prepared based on the composition reported by Alcántara *et al*. for culturing *L. paracasei* BL23 strain [27]. l-Lysine and l-threonine were added at 0.25 g l^−1^ to this medium, whereas leucine was excluded from the composition. Leucine or leucine-containing peptides were added to CDM at a final concentration of 100 µM, and 100 µl of the mixture was dispensed into each well of a 96-well plate. Leucine-containing peptides were selected based on sequences described by Tynkkynen *et al*. [9] and included Leu-Gly, Leu-Gly-Gly and Tyr-Gly-Gly-Phe-Leu (Leu-enkephalin), which were purchased from Peptide Institute, Inc. (Osaka, Japan). Additional peptides, including Gly-Leu-Gly-Lys, Tyr-Gly-Gly-Phe-Leu-Lys (Leu-enkephalin-Lys), Ser-Ile-Gly-Ser-Leu-Ala-Lys and Val-His-Leu-Thr-Pro-Val-Gly-Lys, were synthesized by Eurofins Genomics Inc. (Tokyo, Japan) with a purity of >95 %. Bacteria cultured for 24 h in MRS medium were diluted tenfold in 0.85% (w/v) saline, and 1% (v/v) of the diluted mixture was added to CDM (Leu−) containing each peptide. The cultures were incubated at 37 °C for 48 h. Turbidity at OD_600_ was measured using a SpectraMax M2 plate reader (Molecular Devices LLC, CA, USA).

### Statistical analysis

All statistical analyses were performed using R version 4.4.1 [28]. For selected pairwise comparisons of interest – that is, specific pairs of data points chosen for statistical comparison based on the experimental design and research questions – Welch’s t-tests were performed, and *P*-values were adjusted using the Bonferroni correction to account for multiple testing. To compare each group with a designated control, one-way ANOVA was conducted using the aov function, followed by Dunnett’s post hoc test using the glht function in the ‘multcomp’ package [29], with the mcp method set to ‘Dunnett’. All visualizations were generated using the ‘ggplot2’ package [30] or Microsoft PowerPoint.

## Results

### Search for oligopeptide transporter candidate genes encoded in the LcS genome

**Fig. 1.**
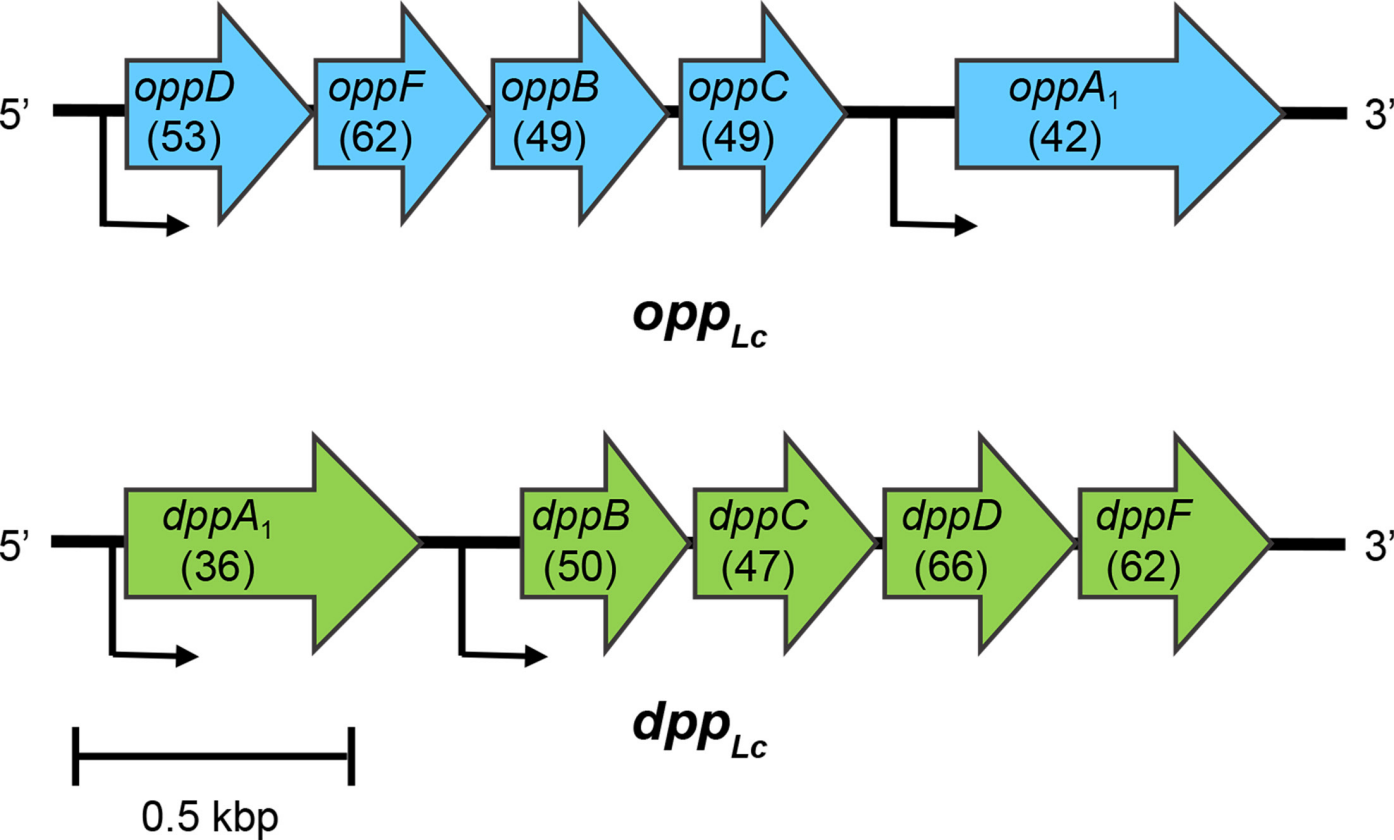
Genetic organization of the *opp_Lc_* and *dpp_Lc_* operons in LcS. The predicted promoters are indicated using arrows. Numbers in parentheses indicate the percentage of identical aa residues in the amino sequence via sequence comparison with *opp* (accession no. L18760) or *dpp* (accession no. AF247635) of *L. lactis*.

To identify the oligopeptide transporters important for LcS growth in milk, we investigated the presence of Opp or Dpp systems in LcS by performing a homology search using *L. lactis* sequences as references [9,10]. Two operons, *opp* and *dpp*, encoding oligopeptide transporters, were identified in LcS (Fig. 1). The *opp* operon gene of LcS (*opp*_*Lc*_) was located on a complementary strand in the order *oppDFBCA*_*1*_, with each subunit showing high identity to the corresponding aa sequences in the *L. lactis* Opp transport system: 53%, 62%, 49%, 49% and 42%, respectively (Fig. 1). Similarly, the dpp operon gene (*dpp*_*Lc*_) was also encoded in the order *dppA*_*1*_*BCDF*, with each subunit showing high homology to the *L. lactis* Dpp system at 36%, 50%, 47%, 66% and 62%, respectively, demonstrating high similarity in all subunits, as observed in *opp*_*Lc*_ (Fig. 1). In both LcS transport systems, *oppD* and *oppF* in *opp*_*Lc*_ and *dppF* and *dppD* in *dpp*_*Lc*_ contain highly conserved ATP-binding domains across different species and show higher homology than other subunits. Additionally, promoters were predicted at two locations in each operon: one upstream of the substrate-binding protein genes (*oppA*_*1*_ and *dppA*_*1*_) and another upstream of the remaining four genes in each operon (Fig. 1)*.*

### Growth of KO strains lacking *opp_Lc_* and *dpp_Lc_* in milk

**Fig. 2.**
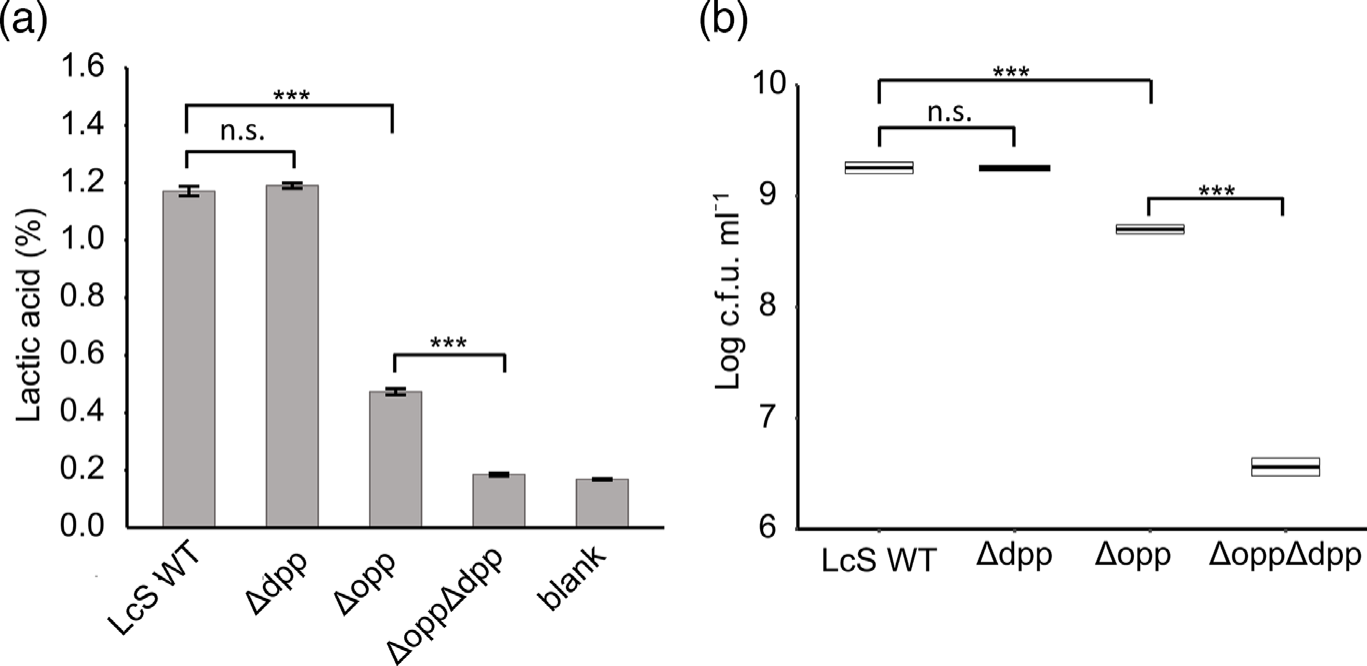
Growth characteristics of *opp_Lc_* and *dpp_Lc_* KO strains. (**a, b**) Viable cell counts (**a**) and titratable acidity (**b**) of LcS WT, KO strains and a complemented strain after 48 h of growth in milk medium. Error bars indicate sd (*n*=3) for each graph. Only comparisons of interest are indicated with brackets. ***P<0.001, n.s. assessed using Welch’s t-test with Bonferroni correction. ∆*opp*, KO strain lacking *opp_Lc_*; Δ*dpp*, KO strain lacking *dpp_Lc_*; Δ*opp*Δ*dpp*, KO strain lacking *opp_Lc_* and *dpp_Lc_*.

Investigating the functional significance of Opp_Lc_ and Dpp_Lc_ in LcS grown in milk is crucial. To this end, we generated three KO strains, Δ*opp*, Δ*dpp* and Δ*opp*Δ*dpp*, in which *opp_Lc_*, *dpp_Lc_* or both operons were genetically disrupted and compared their growth performance in milk with that of WT strains. The viable cell counts and titration acidity of the three test strains and the WT strain cultured in MRS medium were as follows: all strains exhibited viable cell counts ranging from 1.6×10⁹ to 2.1×10⁹ c.f.u. ml^−1^ and titration acidity values between 1.44 and 1.49%, which were comparable to those of the WT strain (2.0×10⁹ c.f.u. ml^−1^ and 1.46%, respectively) (Fig. S2). These findings indicate that all strains grew sufficiently under the preculture conditions. These parameters were subsequently measured in the samples after inoculating the cultures into milk medium. The viable cell count of the Δ*dpp* strain (1.8×10^9^ c.f.u. ml^−1^) was comparable to that of the LcS WT strain (2.9×10^9^ c.f.u. ml^−1^). In contrast, Δ*opp* and Δ*opp*Δ*dpp* strains showed ~5.8-fold and 793-fold lower counts than LcS WT, at 5.0×10^8^ and 3.7×10^6^ c.f.u. ml^−1^, respectively (Fig. 2a). The titration acidity also showed a similar trend, with 1.06 and 1.19% in LcS WT and Δ*dpp* strains, respectively, whereas it was reduced in Δ*opp* and Δ*opp*Δ*dpp* strains (0.41 and 0.19 %, respectively) (Fig. 2b). The average acidity of the milk medium without the inoculum was 0.17% (Fig. 2a). The initial bacterial count was ~1×10^6^ c.f.u. ml^−1^. The Δ*opp*Δ*dpp* strain showed almost no growth and produced no acid during the 48-h incubation period. To confirm that the reduced proliferation of Δ*opp* in milk medium was due to the loss of *opp_Lc_* function, a genetic complementation strain, Δ*opp^c^*, was generated and grown under the same conditions. Δ*opp^c^* demonstrated a significant recovery in viability, with its viable cell count reaching nearly the same level as that of LcS WT (Fig. S3A). The titration acidity of Δ*opp^c^* was marginally lower than that of LcS WT but was notably higher than that of Δ*opp* (0.58%) (Fig. S3B).

### Characteristics of peptide lengths transported by KO strains *opp_Lc_* and *dpp_Lc_*

**Fig. 3.**
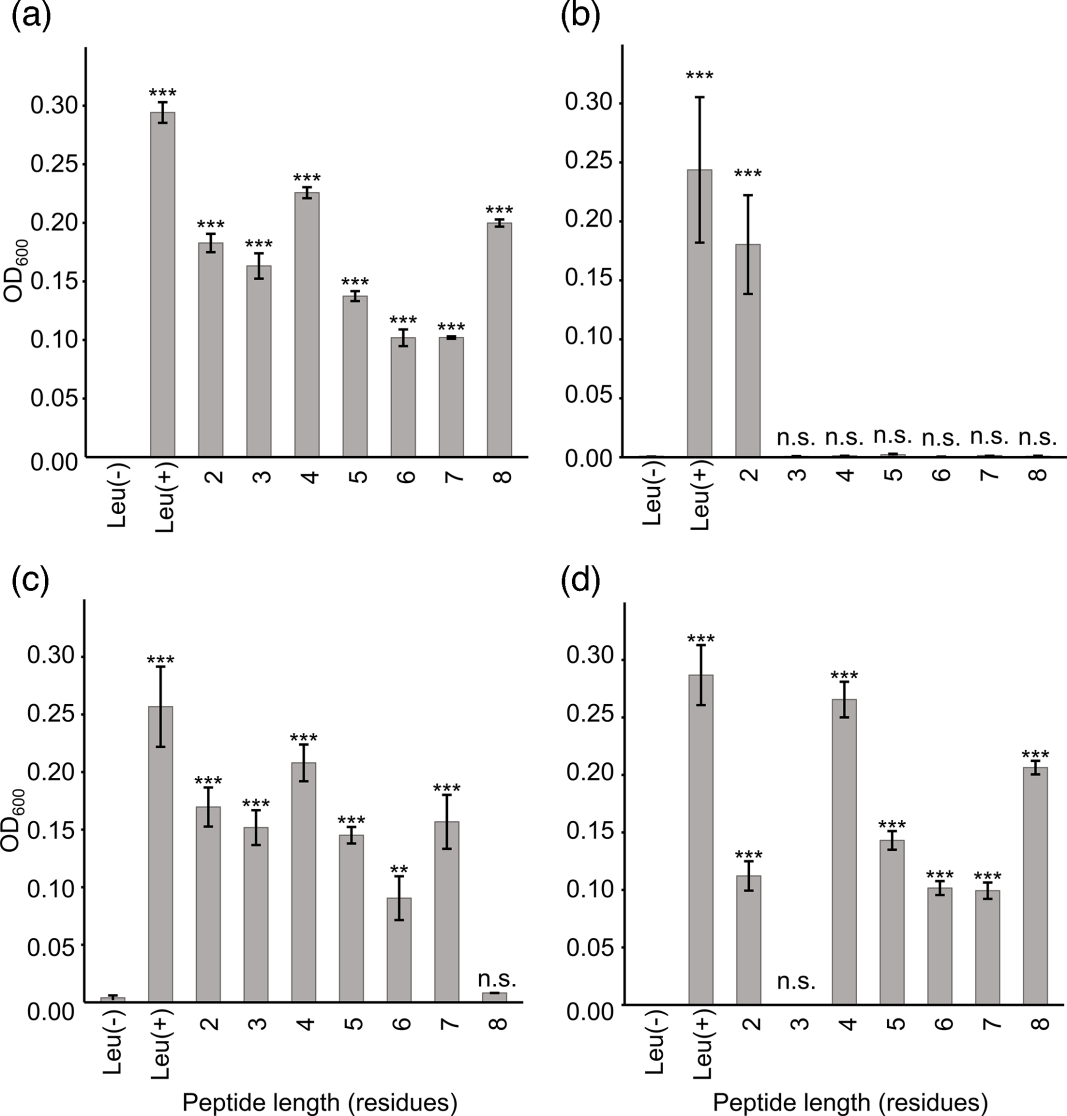
Peptide transport ability of *opp_Lc_* and *dpp_Lc_* KO strains. WT LcS (LcS WT) and gene KO strains were grown for 48 h in CDM lacking leucine and supplemented with a leucine-containing peptide. (**a**) LcS WT; (**b**) Δ*opp*Δ*dpp*, KO strain lacking *opp_Lc_* and *dpp_Lc_*; (**c**) Δ*opp*, KO strain lacking *opp_Lc_*; (**d**) Δ*dpp*, KO strain lacking *dpp_Lc_*. Error bars indicate sd (*n*=2) for each graph. Data represent the means of two parallel experiments. Asterisks indicate significant differences from the Leu(-) group assessed using Dunnett’s test. ***P<0.001, **P<0.01 and n.s., *P*≥0.1. Δ*opp*Δ*dpp*, KO strain lacking *opp_Lc_* and *dpp_Lc_*; Δ*opp*, KO strain lacking *opp_Lc_*; Δ*dpp*, KO strain lacking *dpp_Lc_*.

As LcS is leucine auxotrophic, it cannot grow under leucine-deficient conditions [2]. Therefore, we grew the WT as well as the mutants in media supplemented with peptides of different lengths to assess whether functional differences between the two proposed oligopeptide transporters, Opp_Lc_ and Dpp_Lc_, contribute to growth in milk. We investigated the peptide lengths transported by these oligopeptide transporters. LcS WT grew under all conditions when peptides consisting of 2–8 residues were added (Fig. 3a). In contrast, the deletion of both *opp_Lc_* and *dpp_Lc_*, referred to as Δ*opp*Δ*dpp*, resulted in no growth when peptides containing 3–8 residues were added (Fig. 3b), suggesting additional pathways are involved in transporting 2-residue peptides. A similar analysis with Δ*opp* showed growth in the presence of 2–7 residue peptides but not with 8-residue peptides (Fig. 3c). For Dpp_Lc_, Δ*dpp* grew with the addition of 2 or 4–8 residue peptides but not with 3-residue peptides (Fig. 3d). These findings suggest that Opp_Lc_ and Dpp_Lc_ are involved in the uptake of 4–8- and 3–7-residue peptides, respectively, under the tested conditions. Specifically, Dpp_Lc_ and Opp_Lc_ were responsible for the uptake of the 3- and 8-residue peptides, respectively, whereas the 4–7-residue peptides were taken up by both oligopeptide transporters.

### Growth of KO strains lacking *oppA_1_* and *dppA_1_*

**Fig. 4.**
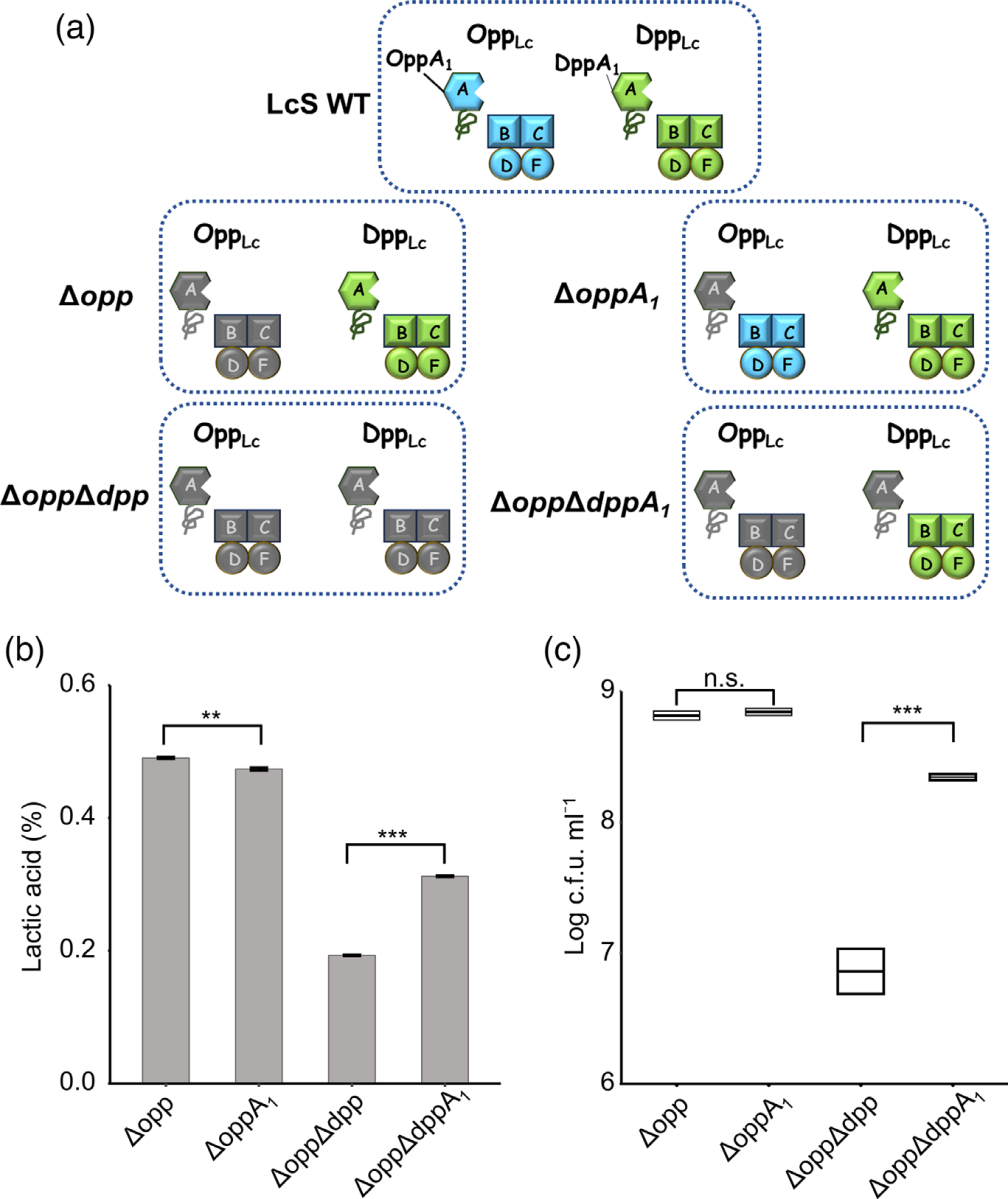
Growth characteristics of KO strains of OBP. (**a**) Schematic representation of Opp_Lc_ and Dpp_Lc_ expression in each strain. Defective proteins in each gene KO strain are indicated in grey. (**b, c**) Titration acidity (**b**) and viable cell counts (**c**) of KO strains after 48 h of growth in milk. Error bars indicate the sd (*n*=3) for each graph. Only comparisons of interest are indicated with brackets. ***P<0.001, **P<0.01, n.s., *P*≥0.1 assessed using Welch’s t-test with Bonferroni correction. ∆*opp*, KO strain lacking *opp_Lc_*; Δ*oppA_1_*, KO strain lacking *oppA_1_*; Δ*opp*Δ*dpp*, KO strain lacking *opp_Lc_* and *dpp_Lc_*; Δ*opp*Δ*dppA_1_*, KO strain lacking *opp_Lc_* and *dppA_1_*.

To identify the OBPs that constitute Opp_Lc_ and Dpp_Lc_ and determine if OBPs other than OppA_1_ and DppA_1_ function as Opp_Lc_ and Dpp_Lc_ subunits, we generated *oppA_1_* and *dppA_1_* gene disruption strains Δ*oppA_1_* and Δ*opp*Δ*dppA_1_* and examined their peptide transport capacity (Fig. 4a). In the absence of functional OBPs other than OppA_1_ or DppA_1_, Δ*oppA_1_* and Δ*opp*Δ*dppA_1_* were expected to completely lose their peptide transport ability via OppA_1_ or DppA_1_, resulting in growth potential equivalent to that of oligopeptide transporter deletion strains, Δ*opp* and Δ*opp*Δ*dpp*, respectively. Although a significant difference in titration acidity was observed between the two strains (estimated difference: 0.0167, 95% confidence interval: 0.012–0.021), no significant difference was found in viable cell counts, suggesting that the acidity difference is not biologically meaningful. In contrast, the titration acidity and viable cell count of Δ*opp*Δ*dppA_1_* were significantly higher than those of Δ*opp*Δ*dpp* by ~0.2% and 29 times, respectively (Fig. 4b, c).

### Characteristics of peptide lengths transported by KO strains lacking *oppA_1_* or *dppA_1_*

**Fig. 5.**
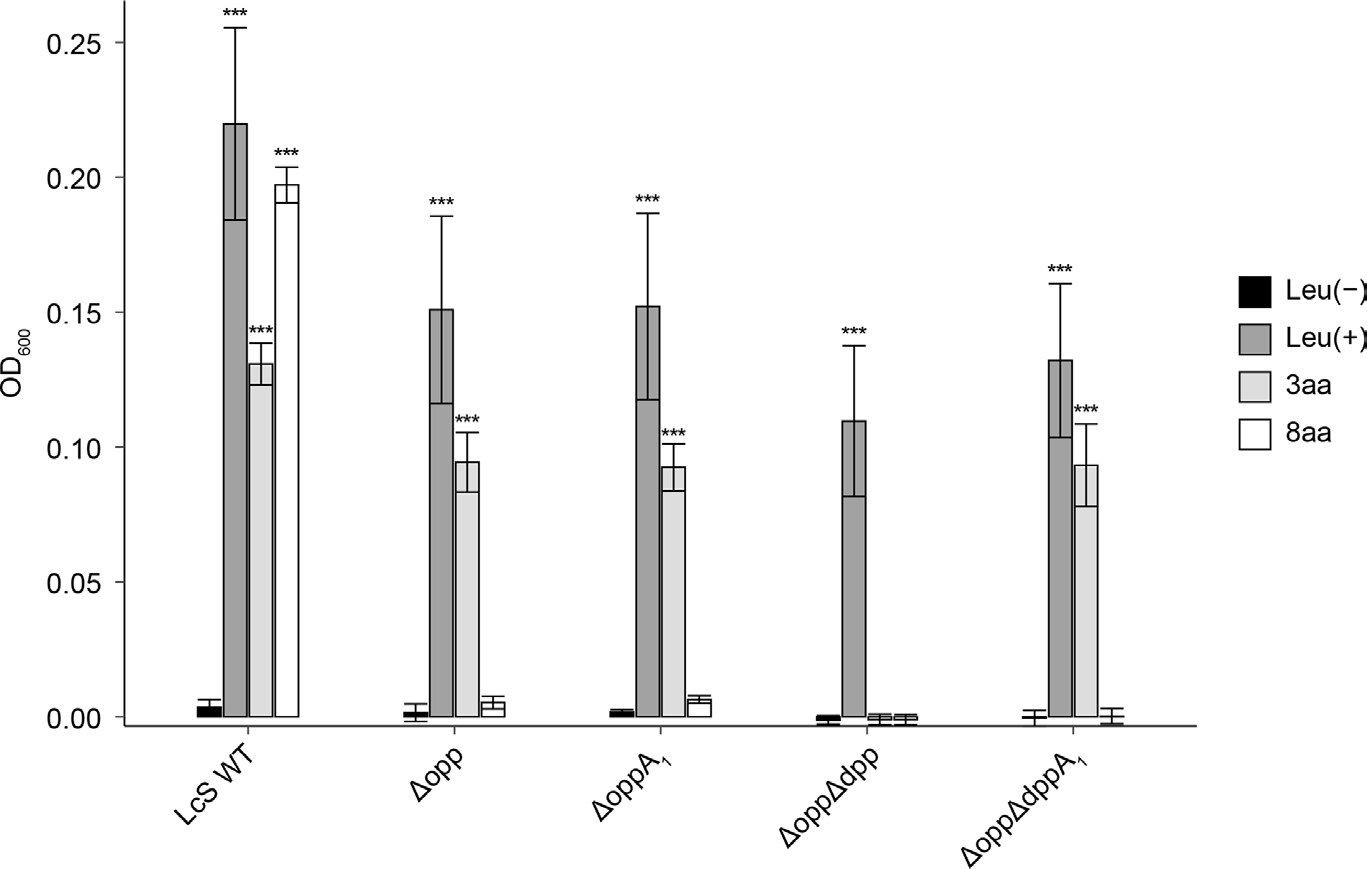
Peptide transport ability of *oppA_1_* and *dppA_1_* KO strains. WT LcS (LcS WT) and KO strains were cultured for 48 h in CDM (Leu−) and supplemented with leucine-containing peptide. Error bars indicate sd (*n*=3). Asterisks indicate significant differences from the Leu(-) group assessed using Dunnett’s test; ***P<0.001. ∆*opp*, gene KO strain lacking *opp_Lc_* genes; Δ*oppA_1_*, KO strain lacking *oppA_1_* gene; Δ*opp*Δ*dpp*, KO strain lacking *opp_Lc_* and *dpp_Lc_* genes; Δ*opp*Δ*dppA_1_*, KO strain lacking *opp_Lc_* and *dppA_1_* genes.

To determine whether OBPs other than OppA_1_ and DppA_1_ play a role in Opp_Lc_ and Dpp_Lc_ function, we compared the characteristics of the transported peptides in the OBP-deletion strains, Δ*oppA_1_* and Δ*opp*Δ*dppA_1_*, with the oligopeptide transporter KO strains, Opp_Lc_ and Dpp_Lc_, respectively. In this analysis, we focused on the ability to incorporate 3- or 8-residue peptides containing leucine, which are considered responsible for the transport functions of Opp_Lc_ and Dpp_Lc_, respectively. Uptake of these peptides into each strain using [CDM (Leu−)] revealed that Δ*oppA_1_*, similar to Δ*opp*, could not grow when eight residues were present (Fig. 5). In other words, both *opp_Lc_*-deficient (Δ*opp*) and *oppA_1_*-deficient (Δ*oppA_1_*) strains lost their ability to take up 8-residue peptides. Conversely, the Δ*opp*Δ*dppA_1_* strain grew when three residues were present, whereas the Δ*opp*Δ*dpp* strain was unable to do so (Fig. 5). These findings suggest that the Δ*opp*Δ*dppA_1_* strain differs from the Δ*opp*Δ*dpp* strain in its ability to incorporate these 3-residue peptides.

### Growth of KO strains in milk compared to OBP candidate gene disruption strains (other than DppA_1_)

**Fig. 6.**
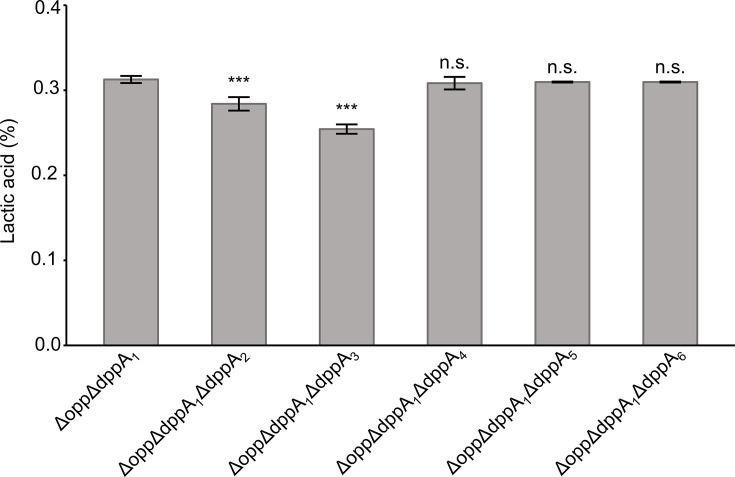
Growth characteristics of candidate gene-disrupted strains of Dpp_Lc_. Titration of acidity after 48 h of growth in milk medium. Error bars indicate sd (*n*=3). Asterisks indicate significant differences from the Δ*opp*Δ*dppA_1_* group assessed using Dunnett’s test; ***P<0.001, n.s., *P*≥0.1. ∆*opp*, KO strain lacking *opp_Lc_* genes; Δ*opp*Δ*dpp*, KO strain lacking *opp_Lc_* and *dpp_Lc_* genes; Δ*opp*Δ*dppA_1_*, KO strain lacking *opp_Lc_* and *dppA_1_* genes; Δ*opp*Δ*dppA_2_*, KO strain lacking *opp_Lc_* and *dppA_2_* genes; Δ*opp*Δ*dppA_3_*, KO strain lacking *opp_Lc_* and *dppA_3_*; Δ*opp*Δ*dppA_4_*, KO strain lacking *opp_Lc_* and *dppA_4_*; Δ*opp*Δ*dppA_5_*, KO strain lacking *opp_Lc_* and *dppA_5_*; Δ*opp*Δ*dppA_6_*, KO strain lacking *opp_Lc_* and *dppA_6_*.

**Fig. 7.**
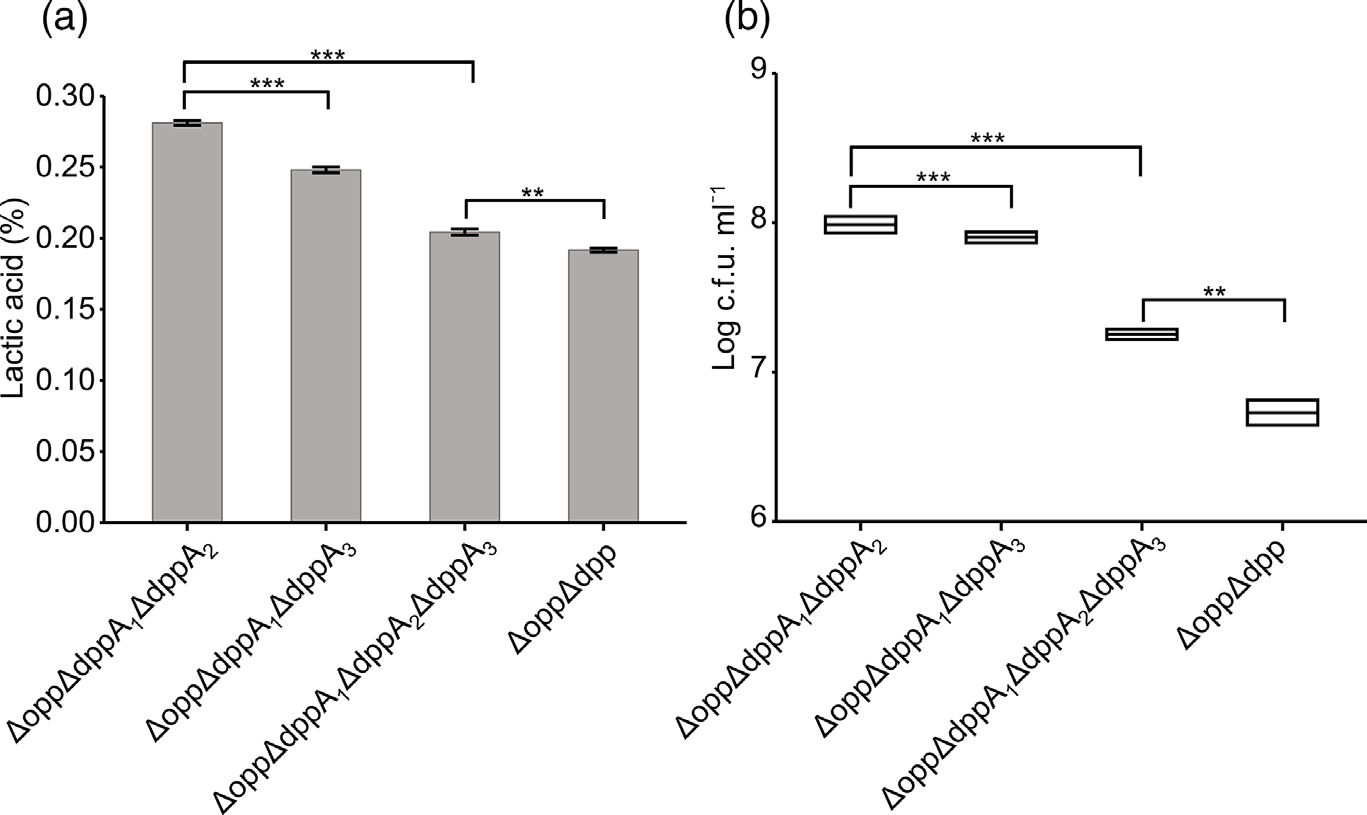
Growth characteristics of KO strains lacking DppA_1_, DppA_2_ or DppA_3_. Titration acidity (**a**) and viable cell count (**b**) after 48 h of growth in milk. Error bars indicate the sd (*n*=3) for each graph. Only comparisons of interest are indicated with brackets. ***P<0.001, **P<0.01. Δ*opp*Δ*dpp*, gene KO strain lacking *opp_Lc_* and *dpp_Lc_*; Δ*opp*Δ*dppA_1_*Δ*dppA_2_*, KO strain lacking *opp_Lc_, dppA_1_* and *dppA_2_*; Δ*oppΔdppA_1_*Δ*dppA_3_*, KO strain lacking *opp_Lc_*, *dppA_1_* and *dppA_3_*; Δ*opp*Δ*dppA_1_*Δ*dppA_2_*Δ*dppA_3_*, KO strain lacking *opp_Lc_*, *dppA_1_, dppA_2_* and *dppA_3_*.

As Dpp_Lc_ may have a functional subunit other than DppA_1_, we searched the LcS genome for genes predicted to encode OBPs and examined their homology with DppA_1_. All genes encoding potential OBP subunits for Dpp_Lc_, referred to as DppA_2_ to DppA_6_, were independently encoded and did not share an operon structure with other oligopeptide transporter genes (Fig. S4). We generated KO strains for five candidate DppA proteins and investigated the titration acidity of each strain after growth in milk. The function of Dpp_Lc_ can only be displayed when Opp_Lc_ is nonfunctional. These five *dppA* gene disruption strains were generated using the Δ*opp* strain as the host organism. The results showed that the titration acidity of Δ*opp*Δ*dppA_1_*Δ*dppA_4_*, Δ*opp*Δ*dppA_1_*Δ*dppA_5_* and Δ*opp*Δ*dppA_1_*Δ*dppA_6_*, targeting DppA_4_, DppD_5_ and DppA_6_, respectively, did not differ significantly compared to that of Δ*opp*Δ*dppA_1_*. However, the titration acidity of the two gene disruption strains Δ*opp*Δ*dppA_1_*Δ*dppA_2_* and Δ*opp*Δ*dppA_1_*Δ*dppA_3_*, which target DppA_2_ and DppA_3_, respectively, was significantly lower than that of the Δ*opp*Δ*dppA_1_* strain targeting DppA_1_. The difference in titration acidity between these two strains with Δ*opp*Δ*dppA_1_* was 0.03% and 0.06%, respectively (Fig. 6). Furthermore, we conducted the same analysis with the Δ*opp*Δ*dppA_1_*Δ*dppA_2_*Δ*dppA_3_* strain, where *dppA_2_*, *dppA_3_* and *dppA_1_* were deleted simultaneously, which showed worse growth in milk than the Δ*opp*Δ*dppA_1_*Δ*dppA_2_* and Δ*opp*Δ*dppA_1_*Δ*dppA_3_* strains. Both titration acidity and viable cell counts in strains Δ*opp*Δ*dppA_1_*Δ*dppA_2_*Δ*dppA_3_* and Δ*opp*Δ*dppA_1_*Δ*dppA_3_* were significantly lower than those in Δ*opp*Δ*dppA_1_* but similar to those in the Δ*opp*Δ*dpp* strain with deficient Dpp_Lc_ function (Fig. 7).

## Discussion

To investigate nitrogen utilization, particularly oligopeptide uptake, in LcS, we analysed the functions of oligopeptide transporters during growth in milk. The LcS genome contains two oligopeptide transporter-encoding operons, Opp_Lc_ and Dpp_Lc_, which exhibit high aa sequence homology with the Opp [9] and Dpp [18] transport systems in *L. lactis*, both of which are crucial for milk-based growth. In LcS, *opp_Lc_* deficiency (Δ*opp*) significantly impaired its proliferation in milk, whereas the simultaneous deletion of *opp_Lc_* and *dpp_Lc_* (Δ*opp*Δ*dpp*) further reduced proliferation, indicating functional redundancy. The partial restoration of titratable acidity observed in Δ*opp^c^*, the *opp_Lc_*-complemented strain, could be attributed to differences between the promoter used in the complementation vector pYAP300 and the native promoter of *opp_Lc_* in the LcS WT strain, particularly in terms of expression timing and strength. Furthermore, Δ*opp*Δ*dpp* also lost the ability to uptake 3–8 residue-long peptides. These findings suggest that Opp_Lc_ and Dpp_Lc_ are crucial oligopeptide transporters for LcS growth in milk, with absorptions of peptides with more than three residues serving as primary nitrogen sources. However, in MRS medium, which is rich in free aa, the Δ*opp*Δ*dpp* strain showed growth comparable to that of the WT strain, indicating that it can proliferate adequately when aa are the primary nitrogen source. In addition, the inability of the Δ*opp*Δ*dpp* strain to proliferate in milk is because of the scarcity of free aa in milk, making it reliant on the absorption of peptides with three or more residues as a sufficient nitrogen source. Our findings indicate that oligopeptides are the primary nitrogen source for LcS growth in milk, consistent with a report that *L. lactis* relies on oligopeptides for more than 90% of its nitrogen requirements during milk-based growth [10].

Our findings also revealed that Opp_Lc_ and Dpp_Lc_ differ in their transportable peptide lengths. Dpp_Lc_ transports peptides with 3–7 residues, whereas Opp_Lc_ transports peptides with 4–8 residues. This overlapping transport range suggests that Opp_Lc_ can compensate for most of the functions of Dpp_Lc_, which may explain why Δ*dpp* showed no significant growth difference in milk compared with the WT strain. However, in this study, only a single peptide sequence was tested for each peptide length, limiting our understanding of how peptide sequence variability affects transport efficiency. Therefore, future studies are required to assess the effect of sequence variation on transporter function.

In *L. lactis*, the proton-coupled transporter, DtpT, is responsible for di- and tripeptide uptake [31]. In this study, we did not investigate *dtpT*. Nevertheless, the observation that simultaneous deletion of both Opp_Lc_ and Dpp_Lc_ (Δ*opp*Δ*dpp*) completely abolished the ability to incorporate 3-residue peptides suggests that DtpT_Lc_ is not involved in the uptake of peptides with three or more residues. However, the possibility that DtpT_Lc_ contributes to dipeptide uptake cannot be excluded, which we intend to explore in future studies. Previous studies in *L. bulgaricus* B14 demonstrated Opp-mediated transport of 5-residue peptides only [19]. In this study, we showed that Opp_Lc_ in LcS can transport peptides up to eight residues, the longest tested peptide, making this the first direct evidence demonstrating the function of oligopeptide transporters in transporting peptides longer than six residues in lactobacilli. However, the ability of Opp_Lc_ to transport >8-residue-long peptides remains to be explored.

These findings align with those of previous studies on the Opp system in *L. lactis* MG1363, where Opp does not import 3-residue peptides but does facilitate the uptake of 4–8-residue peptides [9]. The functional similarity between Opp_Lc_ and *L. lactis* Opp, supported by their aa sequence homology, highlights their analogous roles. Similarly, the capacity of Dpp_Lc_ to transport 3-residue peptides, which Opp_Lc_ cannot, aligns with previous findings [9]. Conversely, the characteristics of the peptides assimilated via Dpp in *L. lactis* vary by strain, depending on the type of OBPs involved. For example, some strains, such as MG1363, express a Dpp optimized for di- and tripeptide uptake, whereas strains like IL1403, SK11, Wg2 and SKM6 possess a second peptide-binding protein, DppP, in addition to DppA, enabling the uptake of 2–9-residue peptides [5]. Our findings confirmed that Dpp_Lc_ exhibited an uptake capacity of up to seven residues, similar to the Dpp type, which is capable of transporting peptides longer than three residues. Nevertheless, further investigation is warranted to determine whether Dpp_Lc_-like oligopeptide transporters function in other *L. paracasei* strains and additional lactobacilli beyond LcS.

Analysis of the OppA_1_ KO strain characteristics revealed that Opp_Lc_ utilizes only one identified functional OBP, OppA_1_, under the tested conditions. This finding suggests that OppA_1_ is likely responsible for transporting 4–8 aa residues. However, the possibility of the existence of other Opp_Lc_-associated OBPs that function under alternative environmental or physiological conditions cannot be ruled out. Therefore, while our data support a central role for OppA_1_ in the tested context, further investigation is required to comprehensively elucidate its substrate range and regulatory mechanisms. In contrast, a comparative analysis of the DppA_1_ and Dpp_Lc_ gene-disruption strains revealed disparities in both proliferation in milk medium and the peptides available for uptake, indicating that Dpp_Lc_ possesses an OBP other than that of DppA_1_. We evaluated the proliferation in milk medium with five candidate genes that were potential OBPs of Dpp_Lc_ and confirmed the functional involvement of two of them, DppA_2_ and DppA_3_. Gene disruption, in which DppA_2_ and DppA_3_ were deleted simultaneously with DppA_1_, showed marginally better proliferation in milk than the gene disruption strain in which the function of the Dpp_Lc_ transport system was completely deleted. This suggests that DppA_2_ and DppA_3_ share certain peptides involved in transport and complement each other’s functions.

Moreover, we showed that *dppA_2_* and *dppA_3_*, which are proposed to be OBPs for Dpp_Lc_, are genetically distant from the *dpp_Lc_* operon. This pattern has also been observed in the Ami transport system, which is analogous to the Opp transport system of *S. thermophilus* [17], and in the Hpp transport system, which is equivalent to the Opp transport system of *Streptococcus gordonii* [32]. Together, these findings suggest that such a genomic configuration is a conserved feature among peptide transport systems in certain LAB species.

Collectively, our findings suggest that two oligopeptide transporters, Opp_Lc_, comprising five subunits OppA_1_BCDF, and Dpp_Lc_, comprising seven subunits DppA_1_A_2_A_3_BCDF, are crucial for nitrogen acquisition in LcS during milk growth. Furthermore, we showed that some oligopeptide transporters found in LcS resembled those in the phylogenetically distantly related *L. lactis*, highlighting the important role of oligopeptide transporters across diverse dairy-related strains. The expression of both transport systems is likely to broaden the range of peptide lengths that LcS can absorb. This phenomenon may represent a survival strategy for LcS, a probiotic lactic acid bacterium used in dairy production, to enhance peptide uptake efficiency, which is vital for nitrogen acquisition in milk. This study provides insights into the characteristics of peptides that Opp_Lc_ and Dpp_Lc_ can transport; however, the analysis was limited to the test peptides of eight residues or fewer. It remains unclear whether transport efficiency is influenced primarily by peptide length or specific aa motifs. Since the test peptides contained conserved residues such as Gly and Leu, future studies should investigate sequence-specific uptake preferences, including peptides of identical length but differing in primary sequence. We plan to conduct a more comprehensive analysis of the peptides taken up by oligopeptide transporters, particularly in the context of LcS growth in milk medium or other fermentation substrates.

## Supplementary material

10.1099/mic.0.001624 Uncited Supplementary Material 1.

## Abbreviations

CDM (Leu−): chemically defined medium lacking leucine
KO: knockout
LAB: lactic acid bacteria
MRS: de Man, Rogosa, Sharpe
OBP: oligopeptide-binding protein
