# Supplementary material for: Identification and functional analysis of two oligopeptide transporters supporting the growth of Lacticaseibacillus paracasei strain Shirota in milk
Source: Microbiology (Reading). 2025 Nov 20;171(11):001624. doi: 10.1099/mic.0.001624 (PMC12634024; doi:10.1099/mic.0.001624)
Supplement: Uncited Supplementary Material 1. [file mic-171-01624-s001.pdf]

Table S1. Sequences of the primers used in this study.

Target gene	Primer	Sequence (5′→3′)	Purpose
opp $_{Lc}$	opp-frag1-F	aatcgtcgacagtcccgataaaatcagccc	Construction of pYSSE3-opp
	opp-frag1-R	gacggtacagagtcgggatgatcataatagtcgtctccga	
	opp-frag2-F	tcggagacgactattatgatcatcccgactctgtaccgtc	
	opp-frag2-R	agtcaagcttgcgtttccgcgccccagctt	
dpp $_{Lc}$	dpp-frag1-F	aatcgtcgacactttttgcccagttgccag	Construction of pYSSE3-dpp
	dpp-frag1-R	gttcattgaagcacatacctttactgctgctagaa	
	dpp-frag2-F	gcagcagtaaaggtatgtgcttcaatgaacgcgta	
	dpp-frag2-R	agtcaagcttgcccgccgtccacgttttaa	
oppA $_1$	oppA$_1$-frag1-F	acccggggatcctctagagacacaaaatcaatctcagt	Construction of pYSSE3-oppA$_1$
	oppA$_1$-frag1-R	acgcggaagggggaaaaagtaagttagatcttacagggca	
	oppA$_1$-frag2-F	gaccataatccgactaagttcctgttccctcctaaaatga	
	oppA$_1$-frag2-R	ctgcaggtcgactctagaccaacattttgttgccattc	
dppA $_1$	dppA$_1$-frag1-F	acccggggatcctctagatggtcagtcccatgataactg	Construction of pYSSE3-dppA$_1$
	dppA$_1$-frag1-R	tcattttaggagggaacaggaacttagtcggattatggtc	
	dppA$_1$-frag2-F	gaccataatccgactaagttcctgttccctcctaaaatga	
	dppA$_1$-frag2-R	ctgcaggtcgactctagaccaacattttgttgccattc	
dppA $_2$	dppA$_2$-frag1-F	acccggggatcctctagaacccaggtattttgacacac	Construction of pYSSE3-dppA$_2$
	dppA$_2$-frag1-R	gtttttgattttgataatattactcaccctcaaaatgat	
	dppA$_2$-frag2-F	atcattttgagggtgagtaatattatcaaaatcaaaaaac	
	dppA$_2$-frag2-R	ctgcaggtcgactctagaaaatttagcctgcggatgca	
dppA $_3$	dppA$_3$-frag1-F	acccggggatcctctagactgccggtgtctgatctggc	Construction of pYSSE3-dppA$_3$
	dppA$_3$-frag1-R	accaaacgaggtgcacaaaatgacaaaagaaagcaggtcg	
	dppA$_3$-frag2-F	cgacctgctttcttttgtcattttgtgcacctcgtttggt	
	dppA$_3$-frag2-R	ctgcaggtcgactctagagcctgagctgcggtcaccat	
dppA $_4$	dppA$_4$ -frag1-F	acccggggatcctctagacgcatcaaacttttggcgat	Construction of pYSSE3-dppA$_4$
	dppA$_4$ -frag1-R	attaagcatgttgttcgtgaatgtattccctccaaaaatc	
	dppA$_4$ -frag2-F	gatttttggagggaatacattcacgaacaacatgcttaat	
	dppA$_4$ -frag2-R	ctgcaggtcgactctagactaggtaaaatggcgcatatatc	
dppA $_5$	dppA$_5$ -frag1-F	acccggggatcctctagagcgcctgtcgtgacaacaga	Construction of pYSSE3-dppA$_5$
	dppA$_5$ -frag1-R	acaaattggaggggcatgatgcgcttcacggcgttttgaa	
	dppA$_5$ -frag2-F	ttcaaaacgccgtgaagcgcatcatgcccctccaatttgt	
	dppA$_5$ -frag2-R	ctgcaggtcgactctagagtcttgtattggcgatcact	
dppA $_6$	dppA$_6$-frag1-F	acccggggatcctctagagtgttcatatcgaactggta	Construction of pYSSE3-dppA$_6$
	dppA$_6$-frag1-R	tgcggaagggagtatacgatttggcacagactaaagggga	
	dppA$_6$-frag2-F	tcccctttagtctgtgccaaatcgtatactcccttccgca	
	dppA$_6$-frag2-R	ctgcaggtcgactctagacttgattttagtatactggg	
opp $_{Lc}$	opp-full-F	acccggggatcctctagaaggacgcaaaggaggagttcgc	Construction of pYAP300-opp
	opp-full-R	ctgcaggtcgactctagattacttagaagcacggctgctgc	
pYSSE3 or pYAP300	pYASSE-F	agaggatccccgggtaccgagct	Linearized vector
	pYASSE-R	agagtcgacctgcaggcatgcaa	

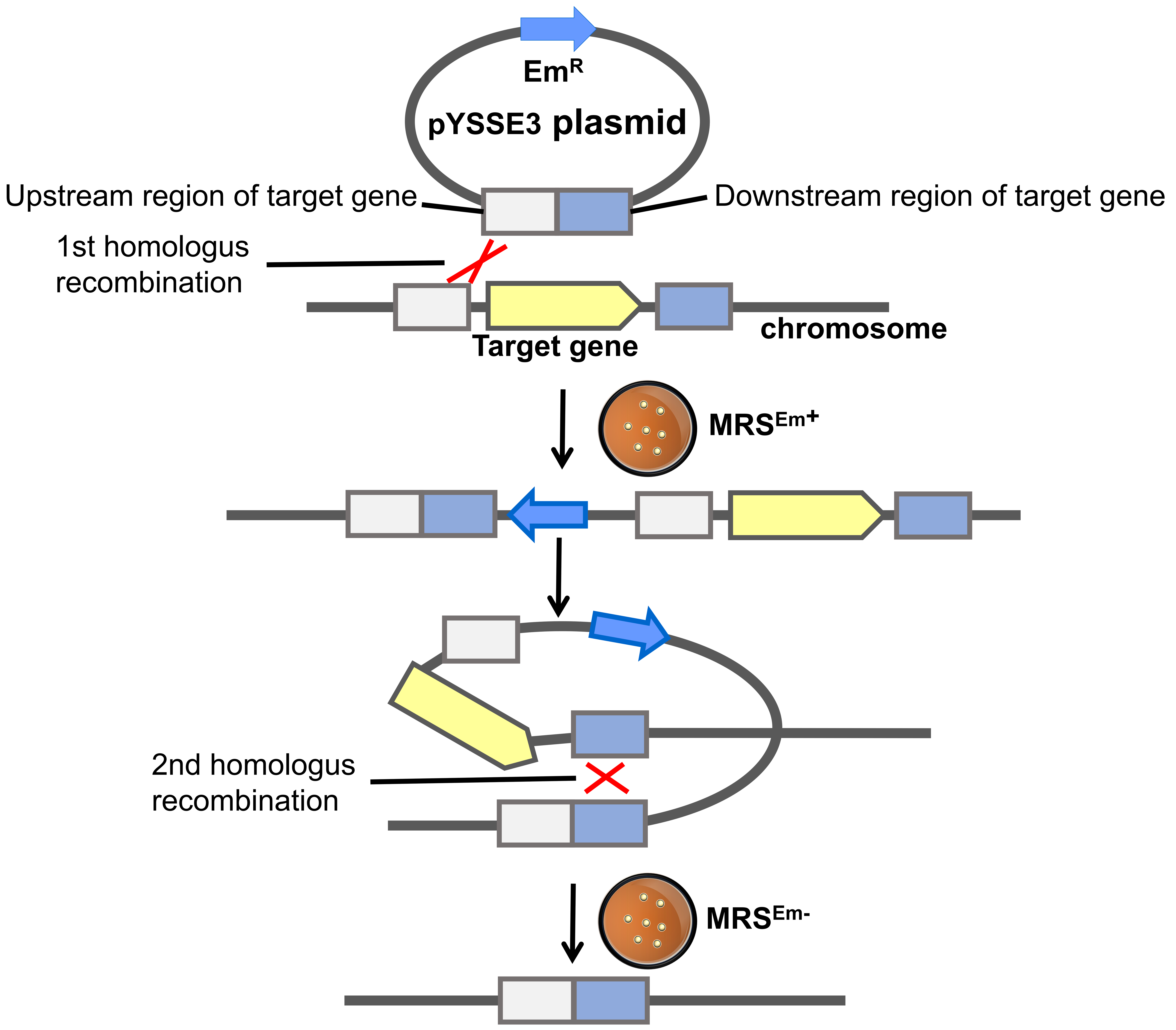

Fig. S1. Schematic diagram of knockout (KO) strain construction. Upstream and downstream regions of the target genes were cloned into the pYSSE3 plasmid and introduced into *Lactobacillus casei* Shirota (LcS) cells. Strains that underwent the first homologous recombination were selected based on growth on erythromycin-containing MRS plates (MRS^{Em+}). Subsequently, strains underwent a second homologous recombination event at a different locus, resulting in excision of the resistance gene based on growth on. Erythromycin-free MRS plates (MRS^{Em-}). The strains were selected based on erythromycin sensitivity. Em^R indicates the erythromycin resistance gene.

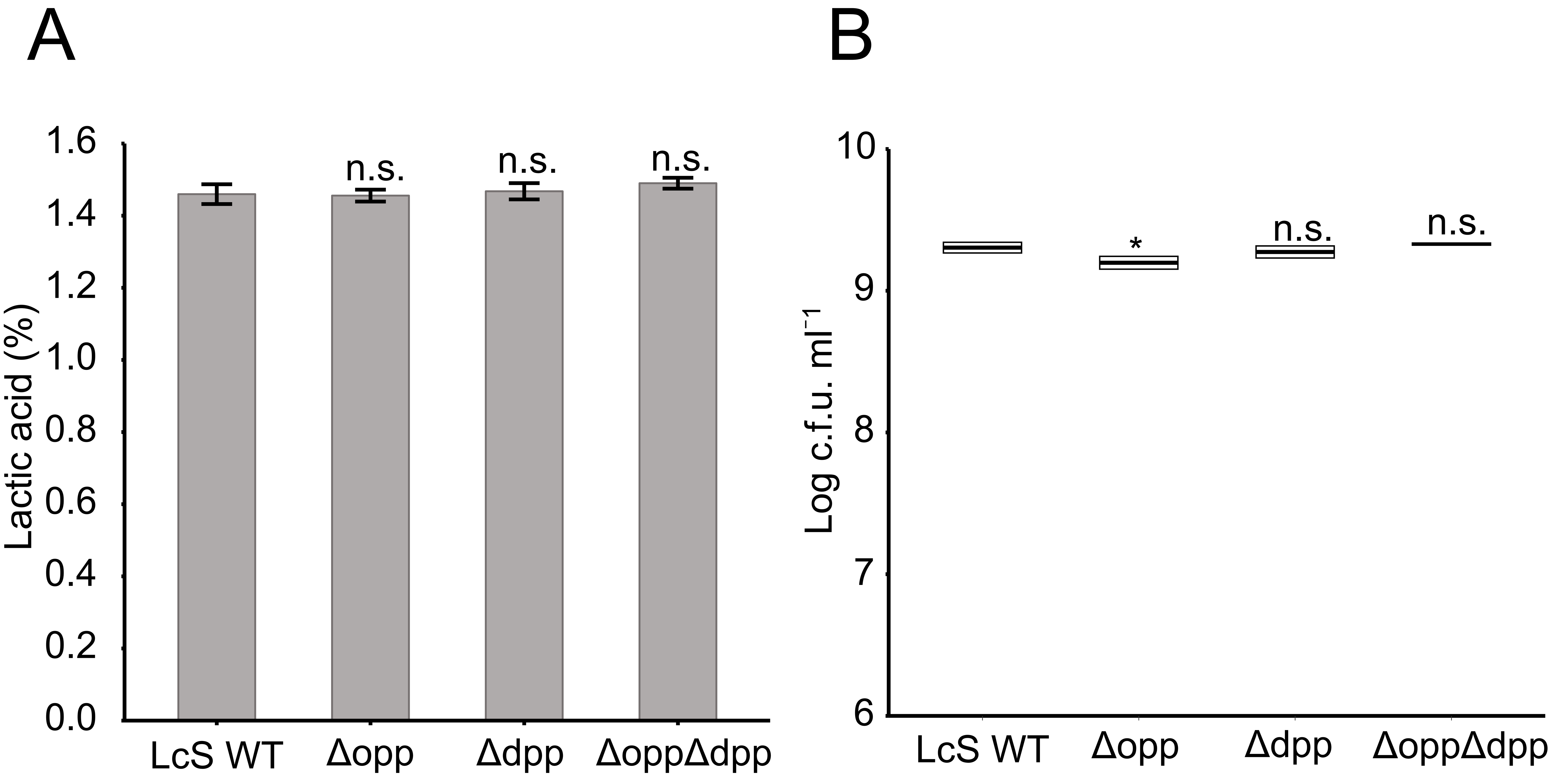

Fig. S2. Growth characteristics of opp_{Lc} and dpp_{Lc} KO strains. (A, B) Viable cell counts (A) and titration acidity (B) of wild-type LcS (LcS WT), gene knockout (KO) strains, and a complemented strain after 24 h of growth in MRS medium. Error bars indicate SD (n = 3) for each graph. Asterisks indicate significant differences from the LcS WT group assessed using Dunnett's test; *p < 0.05, n.s. p ≥ 0.1. Abbreviations: CFU, colony-forming unit; Δ*opp*, KO strain lacking opp_{Lc}; Δ*dpp*, KO strain lacking dpp_{Lc}; Δ*opp*Δ*dpp*, KO strain lacking opp_{Lc} and dpp_{Lc}.

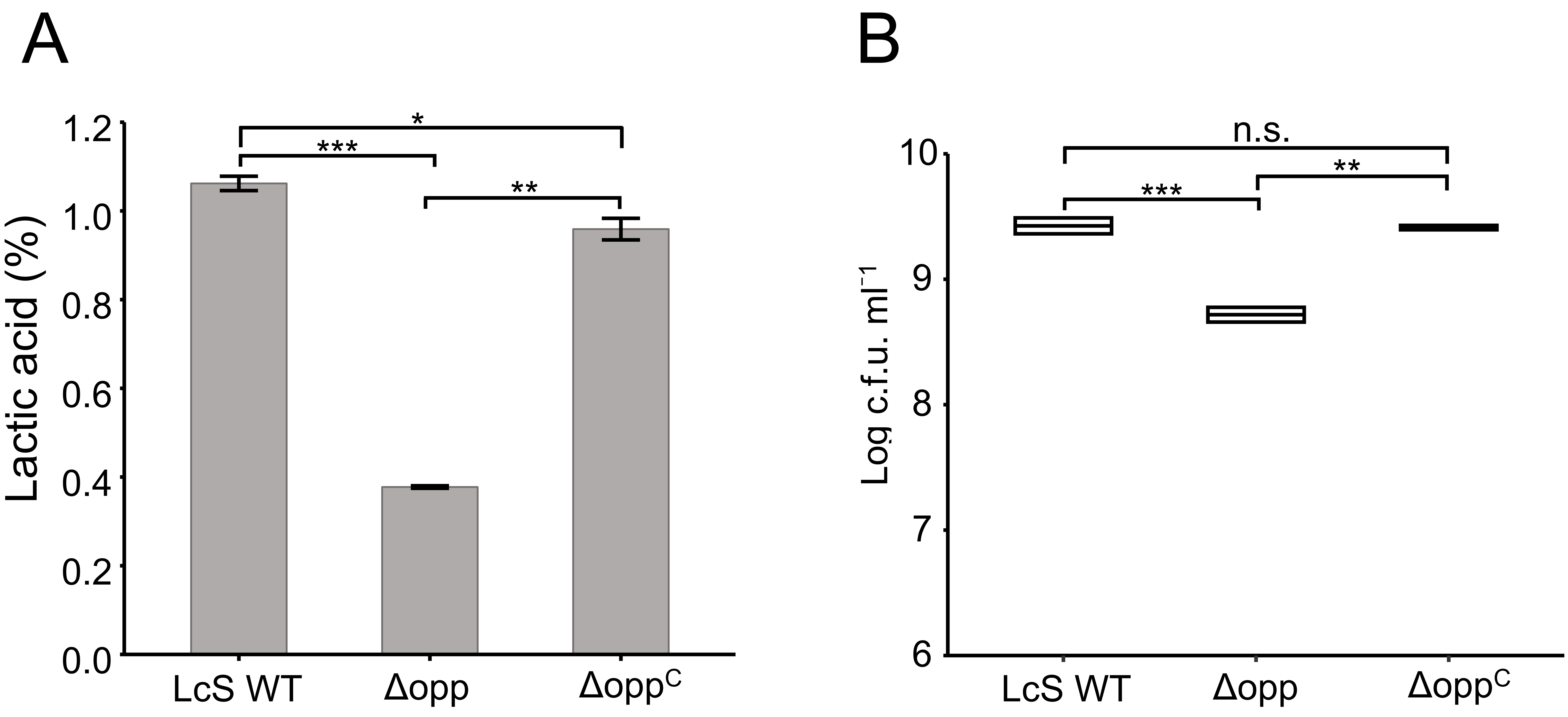

Fig. S3. Growth of the opp_{Lc} complemented strain in milk. Viable cell counts (A) and titration acidity (B) of wild-type LcS (LcS WT), gene knockout (KO) strains, and a complemented strain after 48 h of growth in milk medium. Error bars indicate SD (n = 3) for each graph. ***p < 0.001, **p < 0.01, **p < 0.05, and n.s. for p ≥ 0.1, assessed using Welch's *t*-test with Bonferroni correction. Abbreviations: CFU, colony-forming unit; Δ*opp*, KO strain lacking opp_{Lc}; Δopp^{c}, complemented strain of Δopp_{Lc}.

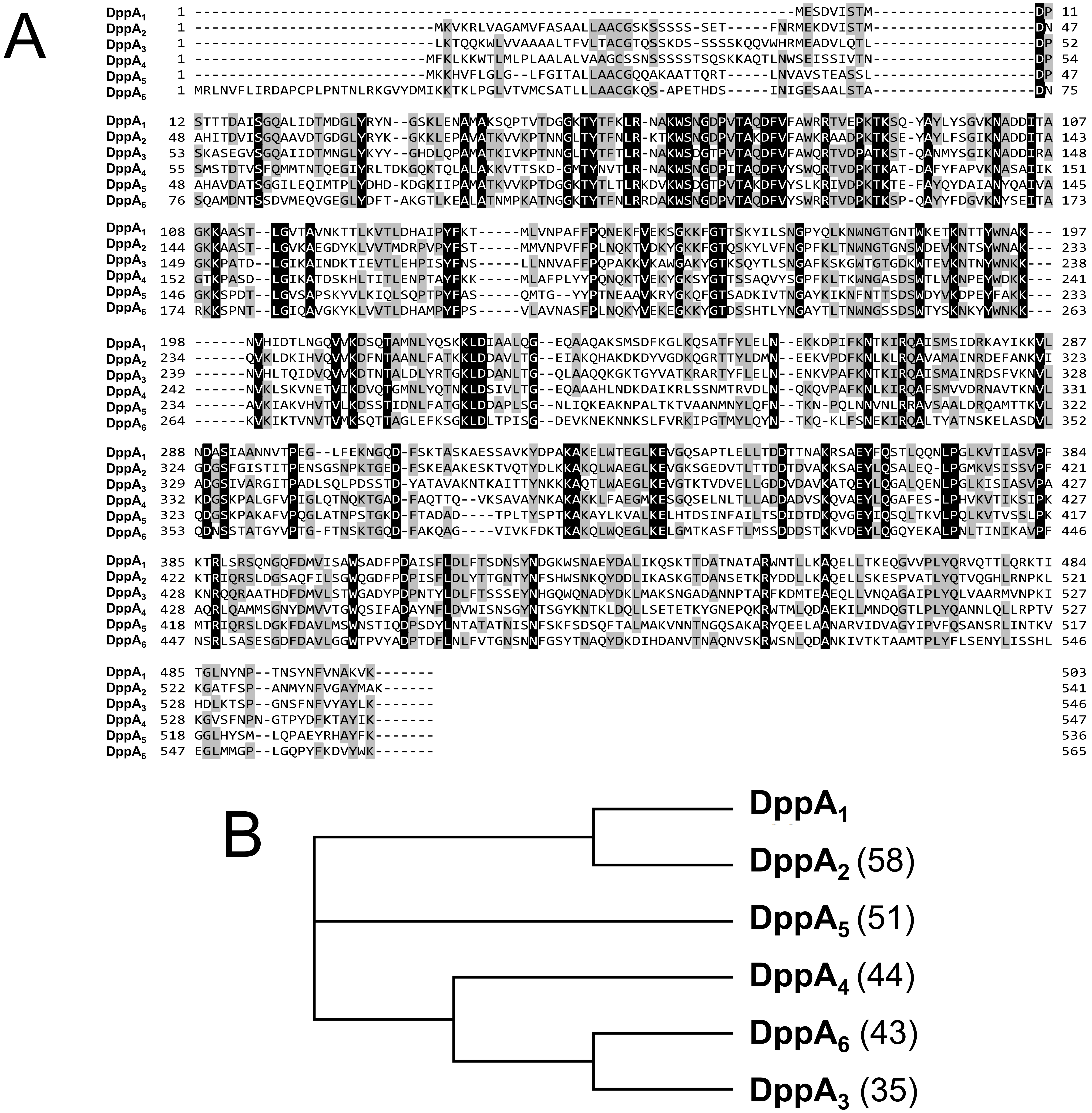

Fig. S4. Sequence similarity of DppA proteins in *Lacticaseibacillus paracasei* strain Shirota (LcS). (A) Multiple alignment of DppA amino acid sequences. Amino acids highlighted with a black background are conserved across all DppA sequences, while those with a gray background are conserved in at least 50% of the sequences. (B) Phylogenetic tree based on amino acid sequence similarity. The numbers in parentheses indicate the percentage identity when aligned 1:1 with $DppA_1$. Accession numbers for each *dppA* are as follows: $dppA_1$: LC855158; $dppA_2$: LC855159; $dppA_3$: LC855160; $dppA_4$: LC855161; $dppA_5$: LC855162; $dppA_6$: LC855163.
